# On the construction of artificial general intelligence based on the correspondence between goals and means

**DOI:** 10.3389/frai.2025.1588726

**Published:** 2025-06-18

**Authors:** Pavel Prudkov

**Affiliations:** Cogsydata.ltd, Jerusalem, Israel

**Keywords:** AGI, goal, means, goal-directed agent, autonomous agent, architecture

## Abstract

Humans are goal-directed agents and intelligence is suggested to be a characteristic of such agents. AGI can be achieved following the principle of the goals-means correspondence that posits the necessary condition for achieving a goal is the correspondence between the goal and the means. The goals-means correspondence is used in all architectures underlying intelligent systems. There are two conventional architectures regarding how the correspondence can be established. One conventional architecture that is based on observations of animals, is intelligent agents whose goals, means, or criteria for its construction are determined jointly at the moment of the birth of an agent. The other conventional architecture that is based on the analysis of human actions, defines intelligent agents whose goals and means are constructed arbitrarily and independently from each other. The conventional architectures cannot explain human actions and thinking. Since the conventional architectures underlie all artificial intelligent systems these systems are insufficient to construct AGI. The formal analysis of architectures demonstrates that there is another architecture in that arbitrary goals and means are constructed jointly on the basis of the criterion of minimal construction costs. This architecture is suggested to underlie human goal-directed processes. The view on humans as goal-directed agents constructing goals and means jointly allows creating an AGI agent that is capable of functioning in real situations. Unlike conventional AI agents that have an unaltered structure, the structure of agents in the new architecture is alterable. The development of an AGI agent may be similar to human growth from an infant to an adult. A model including a simple agent based on the new architecture, is considered. In the model the agent wanders in a quadrangular field filled with various objects that stimulate the agent to move in several directions simultaneously, thus trapping the agent. However, changing its structure the agent constructs goal-directed processes; therefore it is capable of leaving traps.

## 1 Introduction

The appearance of the first computers after World War II inspired outstanding researchers to start considering the construction of computer programs that could think like humans (Turing, 1950). In modern terms, they considered the construction of artificial general intelligence (AGI). The fundamental obstacle for this endeavor was the fact that the general principles and mechanisms of thinking and intelligence were absolutely unclear at that time (and since then the situation has not changed radically). A solution for this problem seemed obvious: it is necessary to simulate some narrow domains of activity where the characteristics of human intelligence are most evident and salient. It was reasonable to assume that certain principles and methods that might be derived from such simulations could be generalized and then artificial general intelligence could be constructed.

However, the history of Artificial Intelligence has demonstrated that the ideas and approaches that could be derived from the construction of intelligent systems for specific domains are not sufficient for the construction of AGI. The simulation of chess playing is an obvious example. Chess playing is often considered the summit of human intelligence. On the other hand, the rules of chess are very simple and unambiguous. As a result, several decades of the simulation of playing chess have led to chess programs that defeat any human player however, those are very specialized and cannot be used beyond chess playing (Levy, 2013). The same situation is observed in other domains related to Artificial Intelligence (pattern recognition, computer games) when artificial intelligent systems compete with or even exceed humans in a specific domain for which those are created; however, such systems are not applicable in other domains.

Modern generative chatbots that are based on large language models (LLMs), are capable of answering arbitrary questions, coding, writing essays (Bang et al., 2023; Lewkowycz et al., 2022). Therefore, one may argue that although LLMs process only discrete information, generative chatbots are not limited to a specific domain. However, LLMs face a fundamental difficulty. Obviously, any intelligent agent interacts with its environment on the basis of a model of the environment. The model of the environment is always simpler than the environment itself. As a result, the efficiency of the agent’s interactions with its environment is limited and the agent is needed to update this model through feedback loops permanently. Accordingly, some components of the model should be more stable than other components, this is necessary to compare the model to the ongoing situation. Human models of the ongoing situation are hierarchical, including both stable and temporary components because human actions are multi-stage. Chess programs have similar characteristics because although the win in a chess game is the stable component of a chess program but the program must flexibly respond to the moves of the opponent through the generation of its own moves. However, LLMs are autoregressive systems that consequentially generate a new item on the basis of previous ones (Lee, 2023); their models of the environment are not hierarchical, therefore, LLMs themselves cannot correct their hallucinations. Generative chatbots imitate some aspects of human intelligence only.

The fundamental distinction between artificial intelligent systems and real systems such as animals and humans, is that artificial systems are adapted to simple artificial environments, but real intelligent systems evolved in the real world that is infinitely complex. On the basis of this distinction, some scholars suggest that AGI cannot be devised at all (Fjelland, 2020).

In this article some general principle that underlies all intelligent systems is considered. Various architectures can be derived from the principle, including those architectures that are usually suggested to underpin human thinking. These conventional architectures are used for the construction of artificial intelligent systems. However, the conventional architectures cannot explain the characteristics of human actions and thinking. A novel architecture is assumed to underlie human thinking. This architecture can be used for the construction of an AGI agent that is capable of functioning in real environments. A tentative design for such an agent is described. In order to demonstrate how the novel architecture can be implemented, a model that includes an agent based on the new architecture is presented.

## 2 The goals-means correspondence

Human beings interact with the world through actions that are directed to the achievement of some results in the future. Thus, humans are goal-directed agents. A goal-directed agent has goals that are the states of the agent or the environment that the agent attempts to achieve or to stay unchanged in the future. It is usually assumed that a goal is the verbal representation of a future state; however, this limitation is unnecessary. Indeed, the behavior of nonhuman animals is directed to the future but they are not verbal. To achieve goals, the agent uses means that are methods for interactions with the world.

I suggest that intelligence is a characteristic of goal-directed agents (Russell and Norvig, 2009). Intelligence reflects the efficiency of the agent in performing actions and achieving goals. From this point of view, humans are intelligent agents that are efficiently able to interact with arbitrary real environments through goal-directed processes. Therefore, in order to create AGI it is necessary to understand how human goal-directed processes are constructed.

Due to the complexity of the world, any intelligent agent has a large reservoir of methods to handle it. However, from the position of internal mechanisms that determine the functioning of the agent, only some of these methods are appropriate to achieve the current goal because such methods somehow correspond to the goal in the ongoing situation. This is the principle of the correspondence between goals and means for the ongoing situation. Of course, this principle is a necessary condition to achieve the goal only. The principle is not sufficient because the world is more complex than the agent; therefore if the agent has an appropriate means, this does not imply *a priori* that the means allows achieving the goal. The establishment of the goals-means correspondence is a necessary prerequisite for the functioning of an intelligent agent in any situation.

### 2.1 Conventional architectures

There may be different architectures for intelligent agents regarding how the correspondence between goals and means can be established. Humans and animals are intelligent agents and observations on their activities allow revealing two architectures. One architecture that it is suggested to underlie the activity of nonhuman animals includes goal-directed agents whose basic goals and means are determined jointly at the moment of the agent’s birth (creation). The actions of such agents result from an activation of innate goals and means or from goals and means that are constructed on the basis of innate criteria for the optimization of the agent’s actions. Innate optimization criteria such as pain, pleasure, or fear define the relationship between the agent and the environment through reward or punishment. Automatic actions, such as habits can also be attributed to this architecture because the result and the method of such an action are activated jointly, and these entities were being constructed regardless of the ongoing situation. Modern intelligent systems based on predetermined criteria of the optimization of a loss function belong to this architecture (Devlin et al., 2018; Silver et al., 2018; Vinyals et al., 2019; Krizhevsky et al., 2017 among others). Undoubtedly, like other animals humans have innate mechanisms associated with survival and reproduction; however, it is obvious that this architecture is unable to explain the diversity and rapid alterations of human actions either at the level of a single individual or at that of a whole society (Buller, 1999).

Our experience teaches us that one goal can be achieved through various means or methods. This, also, implies that one method can be applied to reach different goals. Thus, the analysis of actions and introspection allow us to define the other architecture that includes goal-directed agents whose goals and means can be constructed arbitrarily and independently from each other. It is usually suggested it is the sort of architecture that provides the efficiency and flexibility of human thinking and behavior. Various symbolic systems that were especially popular at early stages of artificial intelligence research can be related to this architecture (Bertino et al., 2001; Jackson, 1998; Newell and Simon, 1972).

If goals are constructed independently from means, then searching through all possible means is the only way to select one or several means that are appropriate to achieve the goal. Since the world is complex, the number of possible means may be very large. Moreover, any possible means itself may result in a new diversity of options and so on. Therefore, with arbitrary goals, searching among possible means can be unrealistically long and expensive. This is the problem of a “combinatorial explosion” of options that was realized by researchers in the 1950s when the first artificial intelligence programs were devised, but its solution has not yet been found (Russell and Norvig, 2009).

Human actions are usually efficient, though people are reluctant to consider many options and they are not being overwhelmed by their decision-making processes. These facts are inconsistent with the suggestion about intensive conscious searching. One may assume that searching among possible means is activated intentionally but it intensively takes place at an unconscious level (Dijksterhuis and Nordgren, 2006). Since the span of consciousness is limited, few means being obtained from searching exceed the threshold of awareness. The most suitable means can be selected among them on the basis of reasoning. As a result, thinking may be efficient without overloading cognitive processes at the conscious level. If this assumption were correct, thinking should be efficient in simple situations such as psychological experiments when the number of possible means is limited; yet, numerous studies reveal that thinking can be remarkably inefficient in such situations (Bruckmaier et al., 2021; Kahneman, 2011; Meyer and Frederick, 2023, among others).

Dual-process models (Evans, 2003, 2008; Kahneman, 2011; Evans and Stanovich, 2013) propose the mind includes two systems. One system, often being designated as System 1, is fast, automatic, and associative. This system corresponds to one architecture described above. System 2 is deliberate, rational, and reflective. Accordingly, System 2 corresponds to the other architecture. This approach suggests that in most everyday situations System 1 automatically selects an appropriate goal and a means on the basis of past experience. Only in rare cases when System 1 fails, System 2 is activated and it provides deliberate searching. According to this approach, thinking may be inefficient in simple situations because automatic processes performed by System 1 are activated in such cases.

However, dual-process models face fundamental challenges. A suggestion that most actions are performed by automatic and associative System 1, is hardly consistent with the general arbitrariness and purposefulness of actions. Indeed, automatic actions that are based on the schemes of past experience only and therefore are inconsistent with the ongoing context, are the distinctive feature of patients suffering from disturbances in the prefrontal cortex (Luria, 2012). Moreover, if there are two systems of thinking then under specific circumstances when it is reasonable to expect the activation of System 1 only (for example, under time pressure), the process of thinking should be qualitatively different from other situations. However, this assumption is inconsistent with experimental data (Bago and De Neys, 2017; Pennycook et al., 2014; Thompson and Johnson, 2014). The conventional architectures cannot explain the diversity and purposefulness of human actions and some characteristics of thinking.

### 2.2 Joint construction of a goal and a means

These two architectures are usually considered two poles of one axis (“automatic” versus “deliberate” or “instinct” versus “intelligence”) and as a result, it seems there are no other architectures. However, a more profound view on the architectures demonstrates that the situation may be more complex. Indeed, the first architecture suggests that the basic goals and means of goal-directed agents are constructed innately and jointly. The second architecture describes agents whose goals and means can be constructed arbitrarily and separately from each other. It is easy to discern that the words “innately” and “separately” are not antonyms; neither are the words “jointly” and “arbitrarily.” This may indicate that the two architectures are only an apparent projection of a two-dimensional structure, in which one dimension can be characterized as “innate” or “predetermined” versus “arbitrary” or “learned” and another dimension as “constructed jointly” versus “constructed separately.” With this assumption, a representation of the structure can be given as Table 1.

**Table 1:** Formal classification of goal-directed architectures.

	Jointly	Separately
Innately	Goals and means are constructed innately and jointly.	Goals and means are constructed innately and separately.
Arbitrarily	Goals and means are constructed arbitrarily and jointly.	Goals and means are constructed arbitrarily and separately.

It is easy to notice that two cells in the table correspond to the conventional architectures but two novel architectures arise from the other cells. One novel architecture is goal-directed agents whose goals and means are constructed innately and separately. This architecture is, however, inconsistent with the principle of the goals-means correspondence. Indeed, since goals and means are constructed separately, they do not correspond to each other, in general. Yet, these entities cannot be modified owing to their innateness.

The other novel architecture is goal-directed agents, whose goals and means are constructed arbitrarily and jointly. It is not difficult to note clear advantages of this architecture. Indeed, because the goal and the means of an agent in this architecture are constructed jointly, there is no need to search among a potentially infinite set of means to satisfy the goal; this is a simple solution for the problem of combinatorial explosion. On the other hand, the possibility to construct goals and means arbitrarily indicates the actions of agents belonging to this architecture may be very flexible and adaptive.

In order to construct a goal and a means jointly, it is necessary to define a criterion that determines its construction. This criterion should be derived from the characteristics of the agent that are not linked to the relationship between the agent and its environment. Indeed, if the relationship underlies such a criterion, then the agent belongs to the architecture with innate goals and means. There may be various internal criteria, but the criterion of minimal construction costs seems very simple and universal. This criterion is that the goal and the means of a goal-directed process are constructed to minimize the costs on its construction. The criterion of minimal construction costs can be considered the transfer from physics to cognitive science, the fundamental principle of least action. In physics, the trajectory of an object is derived by finding the path that minimizes the action (in physics, a quantity that is associated with the energy of the object) (Landau and Lifshitz, 1976).

I posit that humans are goal-directed agents that provide the correspondence between goals and means through their joint construction on the basis of the criterion of minimal construction costs (Prudkov, 2010, 2021; Prudkov and Rodina, 2023). The joint construction of a goal and a means is an absolutely unconscious and uncontrollable process, but it results in the conscious representation of the situation and the individual; that is, the person acknowledges what conscious goal can be achieved in the situation, how this can be performed, and what conscious criteria can be used to evaluate the goal and the means.

The proposition that humans are goal-directed agents whose goals and means are constructed jointly on the basis of the criterion of minimal construction costs elucidates why human actions are flexible and efficient without suffering from the combinatorial explosion of options. However, since thinking is determined by the criterion of minimal construction costs, the result of a thought process may be wrong if the costs associated with incorrect information are minimal. This explains why thinking may be inefficient even in simple situations.

The idea that a goal and a means are constructed jointly is based on the strong evidence that the prefrontal cortex which is responsible for human goal-directed behavior, does not process goals and means separately (Deary et al., 2010; Fuster, 2015).

It is necessary to note that the proposition that the mind constructs a goal and a means jointly does not imply that an individual deliberately cannot search through possible options as a method to determine an appropriate means. Indeed, the conscious thought to apply searching along with the awareness of several possible options may be the result of the ongoing goal-directed process. The criterion of minimal construction costs is a criterion for the construction of actions rather than a criterion for their selection; therefore, the criterion does not imply that people always minimize efforts when they perform actions.

In order to clarify the functioning of joint construction, consider a hungry individual. If she is hungry she may recall some grocery shops and certain information on these grocery shops: routes to it, its prices, and some emotional attitudes regarding the shops. This is an absolutely unconscious joint construction process that results in a coherent representation of the situation and this representation may be appropriate for the satisfaction of the ongoing need. This representation is one among potentially possible others only and it may not be optimal (for example, the grocery shop with minimal prices may not be recalled); however, this is the solution for the problem of the combinatorial explosion. The construction of the model of the current situation is a primary goal-directed process. In principle, one grocery store may be recalled, and then the person may decide to go to this store. When she leaves her home, a new joint construction process arises and results in a new model of the situation when the objects of the environment that may be appropriate for achieving the store become perceptible.

If several grocery stores are recalled then their comparisons and the selection of the best grocery store are necessary. From the position of the joint construction approach (referred to as JCA, hereinafter); these comparisons based on reasoning and planning are secondary goal-directed processes because those are performed within the primary model of the situation. The consequence of such comparisons can be the awareness of the failure of the primary model and then a new primary model emerges. In other words, feedback loops from interactions between the individual and the environment lead to the construction of novel goal-directed processes; however, since practically any action is multilevel, changes at the lower levels of a goal-directed process are possible without changing at its upper levels.

One may propose two obvious objections to the joint construction approach. First, if a goal and a means are constructed jointly, then the means ought to correspond to the goal somehow. However, people often understand what goal must be achieved but they cannot suggest a method to achieve the goal. For example, the author would like to be a winged dragon roaming between stars but he has no idea how to be converted into such a dragon. Yet, a person can dream of becoming a dragon only if she preliminary has selected information about dragons from the infinite variety of information about the world. The formation of a consistent view on a certain aspect of reality is an obvious condition for any activity aimed at this aspect.

The joint construction is not a method to create the best action (this is impossible due to the combinatorial explosion) but a method to create some action (because the number of possible actions is infinite, in principle). To some degree, an alternative to the action that is formed by the ongoing joint construction process is not another action but rather its absence. Therefore, the idea of joint construction is not hurt by the fact that people are able to imagine, plan, or pursue completely arbitrary, even unachievable goals. When the individual thinks that there is no method to achieve the goal, nevertheless an inappropriate method is chosen because the selection of a specific aspect of reality among other possible aspects occurred.

Second, as is pointed out above, one goal can be achieved by various methods and one method can be applied to achieve various goals. These facts seem inconsistent with JCA. The idea that goals and means can be constructed separately is correct at the level of social practice but a psychological illusion at the level of psychological mechanisms of a particular action.

In order to clear this proposition, imagine that one needs to achieve the 35th floor of a skyscraper. Firstly, this can be made by means of an elevator. If no elevator can be used (e.g., there is no voltage), it is possible to go upstairs. Finally, if the staircase is destroyed, then one can climb on the wall using appropriate tools. It seems one invariable goal can be combined with various methods to achieve it. However, the first method is available for everyone because it requires no concentration of mental resources. The second one can be accepted when there is a serious need to reach the goal. The last one can be used only under extreme circumstances requiring the strongest concentration of will and energy. In other words, from the position of internal processes, each way requires a specific psychological arrangement with special goals and means and this arrangement is acknowledged by any individual as distinctive from the others. Therefore, a change in the situation results in the alteration of goals and means at a specific level of the hierarchy of goals. It is reasonable to assume that the interaction between goals and means in the process of the construction of a goal-directed activity is a characteristic of any activity.

The psychological illusion of the separate construction of goals and means results from the fact that it is very difficult to combine the involvement in a particular activity with the simultaneous introspective monitoring of this activity. Indeed, when an individual pursues an everyday goal (e.g., shopping at the supermarket), she usually does not pay attention to all variations in intermediate goals and means that are necessary for this multi-stage pursuit. As a result, the complex interplay of these intermediate processes is reflected by consciousness and memory only partially, while the success or failure in the achievement of the main goal is usually in the focus of consciousness.

Prudkov and Rodina (2023) examined a hypothesis that the joint construction approach determines actions entirely. In the experiment, participants were informed about the joint construction mechanism and instructed to violate its functioning by performing an action. Participants could violate the functioning of the mechanism at two levels of the action but information about one of the levels was more explicit than about the other level. It was assumed that participants would violate the functioning of the mechanism only at one level. This assumption was confirmed experimentally. This indicates that joint construction determines actions because a sort of compliance between these levels was necessary to perform the action. Participants really did not violate the mechanism of joint construction; simply, in the unusual conditions of the experiment, joint construction brought about special results.

## 3 Creation of an AGI agent

The understanding of humans as goal-directed agents whose goals and means are constructed jointly paves the road to the creation of an AGI agent. As is pointed out above, I suggest that an AGI agent is an intelligent agent that is capable of acting in the real world as flexibly and efficiently as human beings.

In order to interact with the world, an intelligent agent is needed to build the internal model of the ongoing situation and then to use the model as the basis for the interaction with the situation. To build the model, it is necessary to divide the ongoing situation into separate objects and relations between them. Since the world is infinitely complex, the division of the situation into separate objects and relations is always relative and it is determined by the needs and goals of the agent; otherwise, these needs and goals cannot be satisfied. Thus, dividing the situation into separate objects is a component of establishing the current goal-means correspondence. It is reasonable to suggest that the search for an efficient way for the building and application of the internal model of an environment through the optimal establishment of the goals-means correspondence is the main objective for research in artificial intelligence (Matsuo et al., 2022).

As is noted above, building the internal model by humans is an instant and unconscious process and only its result is available to consciousness. On the other hand, the interaction with the situation is an effortful conscious activity. As a result, people are intended to neglect the complication of the division of the situation into objects and they preferably pay attention to the importance of some tools, such as reasoning or heuristics that can be used to function in the situation efficiently. This characteristic of human thinking has influenced the advance in the field of AI.

In the 1950s when AI research started, the architecture with the independent and separate construction of a goal and a means underpinned the activity of most researchers and they considered symbolic systems to be intelligent agents. In accordance with the idea on the importance of tools, those researchers suggested that the combinatorial explosion is the main obstacle for the creation of AI. They believed the mitigation of the combinatorial explosion was sufficient to establish the goals-means correspondence regardless of the complexity of the environment; therefore, they used artificial environments with very simple and unequivocal objects such as chess rules and pieces.

In the 1960s various techniques against the combinatorial explosion were proposed and some scientists optimistically wrote that the problem of artificial intelligence will be solved in a generation (Crevier, 1993). When researchers started to use environments with more complex and diverse objects, they attempted to define the required characteristics of such objects “by hand,” that is, by the explicit and deliberate selection of those characteristics and relations that may be appropriate for the efficient establishment of the goals-means correspondence. However, they revealed that this approach did not allow establishing the adequate goals-means correspondence for complex artificial environments. This caused a very long period of stagnation in Artificial Intelligence research (Crevier, 1993; Russell and Norvig, 2009). The failure of symbolic systems can be considered another piece of evidence favoring a notion that the architecture with the independent and separate construction of a goal and a means does not underlie the establishment of the goals-means correspondence in humans.

The contemporary approach considers networks with neuron-like units intelligent agents whose goals and means are constructed together applying a predetermined optimization criterion (or criteria) associated with supervision or reinforcement. This approach describes objects at a sub-symbolic level and, in accordance with the idea of the importance of tools, the main task is considered to be searching for techniques for the “automatic” establishment of the goals-means correspondence as learning in a multi-layer network of neuron-like units. The appearance of such techniques and incredible increase in computer power enable the establishment of the goals-means correspondence for very complex artificial environments and the construction of such agents as generative chatbots.

Such results encourage some researchers to claim that this approach is the solution to artificial general intelligence (Silver et al., 2021). This statement faces two obvious problems. First, the world is infinitely complex and changeable; therefore, it is impossible to establish the goals-means correspondence that could be appropriate for arbitrary goals and situations in advance. Second, although the establishment of the goals-means correspondence itself is an “automatic” process in the approach, various characteristics associated with supervision/reinforcement, the optimization criterion (criteria), and the objects in the environment are defined “by hand”; therefore, generally, these entities are not related to each other. As a result, the establishment of the goals-means correspondence may be a time-consuming and often multi-stage process. Such a process is feasible for artificial systems because artificial systems are entirely controllable. The state of an artificial agent and the state of an artificial environment can be reproduced or changed as many times as necessary; yet, this is not feasible for real situations (Matsuo et al., 2022).

JCA posits that the division of the world into objects and relations is a component of the joint construction of a goal and a means; therefore, a JCA agent theoretically can establish the goals-means correspondence for real environments fast and automatically. JCA suggests that like humans, a JCA agent is capable of developing; therefore, if the agent starts from short-term goals and simple characteristics of objects, then long-lasting goals and means based on the complex characteristics of objects can be achieved gradually through interactions with the world. Such characteristics of human intelligence as reasoning and planning can be the results of gradual changes in the functioning of the agent because, as it is pointed out above, reasoning and planning are also goal-directed processes.

The basic goals of an artificial agent in the conventional architectures, that is, some results that the agent is intended to achieve according to the agent’s creators, are built into the design and structure of the agent directly or through optimization criteria and this determines the agent’s behavior. As is pointed out above, it is difficult to predict how a chess program can play in a concrete game but in any game the program strives to win because this is built into its design. Accordingly, corresponding means are also designed directly or through criteria of optimization. Yet, a goal-directed agent based on JCA has no basic goals and means; in general, its goals and means are arbitrary and should be learned. It is reasonable to assume that a JCA agent is a distributed system such as a neural network. In this case, the goal and the means of a goal-directed process are constructed from some interactions among the elements of the network following the criterion of minimal construction costs. These interactions may increase the coordination and coherence among the elements, for example, by generating novel constituents of the network or eliminating present ones. If the level of coherence exceeds a threshold, then the construction of a goal and a means is completed and a novel goal-directed process starts. The goal and the means are distributed over the changed structure of the agent. The coherent functioning of the network may be stable and persistent; therefore, it may regulate the actions of the agent and direct the agent to its goals. Thus, unlike conventional AI agents that have an unchangeable structure, the structure of JCA agents is alterable.

From this position, the construction of a new goal-directed process on the basis of the criterion of minimal construction costs involves two constituents. One constituent is the minimization of interactions among the elements of the network and the second constituent is the minimization of changes in the structure of the network that are caused by these interactions.

Let us denote the variable structure of the neural network representing the agent at moment t as *Ω*(t). ρ() is a metric of an element of the network in the space of the elements of the network. *Λ* is an operator, that describes interactions among the elements. *Ξ* is an operator that describes changes in the structure.

Then the construction of a new goal-directed process meets the following optimization problem:


(1) $$\underset{\Omega \left(t\right)}{\min}\Phi (\Lambda \left(\rho \left(\left(\Omega \left(t\right)\right)\right),\Xi \left(\Omega \left(t\right)\right)\right)\;giventhat\;\zeta \;\left(\Omega \left(t\right)\right)>T,$$

here *Φ* is an operator; *ζ* () is a coherence function; T is a threshold of coherence.

It is reasonable to assume that the state of the space and the operator of changes in the structure are independent from each other, then Equation (1) can be presented as follows:


(2) $$\underset{\Omega \left(t\right)}{\min}\Lambda (\rho \left(\Omega \left(t\right)\right)+\underset{\Omega \left(t\right)}{\min}\Xi \left(\Omega \left(t\right)\right)\;giventhat\;\zeta \;\left(\Omega \left(t\right)\right)>Τ\;$$

A satisfactory definition for the components of Equation (2) and its optimization is not a trivial problem. The methods of neural architecture search (Stanley et al., 2019; Ren et al., 2021; Parker-Holder et al., 2022) do not seem appropriate for this because these methods enable selecting an agent with an unchangeable structure among other similar agents on the basis of criteria linked to the relationship between the agent and the environment.

It is not simple to define conditions under which a goal-directed process may start or complete because with a changeable structure determined by internal processes, the agent is similar to a black box. Moreover, the agent’s view on the situation may be distinctive from the researcher’s view because different characteristics of the objects may underlie these views. However, a rough estimate of the agent’s state at moment t is obviously possible. One may say that this estimate is similar to human EEG. Let us denote the estimate as *λ*(t). *λ*(t) may be a vector; however, for simplicity, λ(t) is a number. One can present *λ*(t) as follows:


(3) $$\lambda \left(t\right)=\theta \left(H\left(t\right),K\left(t\right)\right),$$

where θ(t) is a function.

Η(t) is an operator that describes the anticipations of the agent based on the current goal regarding the environment at moment t. The higher Η(t), the more the agent expects changes in the environment. Κ(t) is an operator that describes a real state of the environment from the agent’s position at moment t. The higher *Κ*(t), the greater the changes in the environment.

θ(t), *Η*(t), and *Κ*(t) are not available for the researcher. Although Η(t) and *Κ*(t) can be interconnected because the agent could interact with the environment at early moments, in general, the changes in the environment are independent from the agent’s actions. Also, let us assume that θ(t) is a constant, Η(t), and Κ(t) are functions. Then one can approximate Equation (3) as follows:


(4) λ(t)=c∗H(t)∗K(t)

There may be several conclusions from Equation (4). If λ(t) is large, this indicates Η(t) and Κ(t) are large. In this case, a new goal-directed process is needed to respond to changes in the situation. If *λ*(t) is not very large, then the ongoing process is sufficient, although some changes in the structure and state of the agent are possible, for example, when another means is activated. If *λ*(t) is small, this meets three different options: Η(t) and Κ(t) are small; Η(t) is small and Κ(t) is not small; Η(t) is not small and Κ(t) is small. The first two options indicate that the goal has been achieved and the third option indicates the environment is stable. These situations are different; hence, in general, it is difficult to establish a criterion for the achievement of the goal.

These results are related to a simple agent that has one level. Let us consider, that an agent has two levels, level 1 and level 2. Level 2 receives some information from level 1 through feed-forward connections, generalizes the information and then controls level 1 via feedback loops_._ Let us assume that *λ*(t)_1_ and λ(t)_2_ are the estimates of activity at level 1 and level 2, respectively. It is reasonable to suggest that λ(t)_1_ usually is greater than λ(t)_2_. This indicates that there are two active processes and the process at level 2 controls the process at level 1.

If λ(t)_1_ is less than λ(t)_2_ this indicates the achievement of the goal at level 1. It is reasonable to assume that if λ(t)_2_ is small within a long interval of time and λ(t)_1_ is variable at that time, this indicates the achievement of the goal at level 2. This approach can be generalized for agents with many levels.

**Figure 1:**
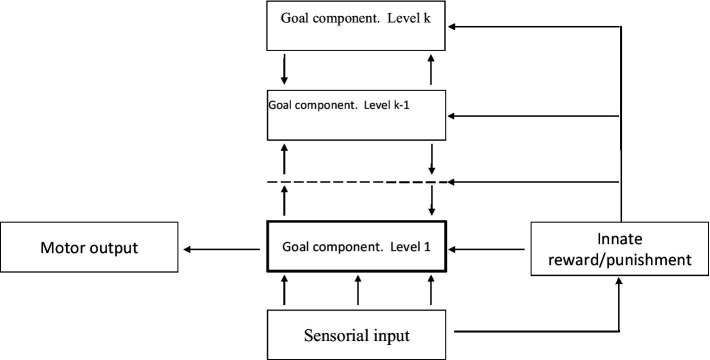
Tentative design of an AGI agent.

It is reasonable to suggest that the development of an AGI agent should be similar to human maturation. An infant is able to perform short-term actions on the basis of sensorial information. The actions of an adult can be a long-term even life-term hierarchical systems based on symbolic representations of future results. Although the mechanism of growing up is poorly understood, it can be assumed that this is the result of a particular architecture in the prefrontal cortex. There is a caudal-rostral gradient in the prefrontal cortex where caudal regions respond to immediate sensory stimuli, middle regions select actions on the basis of prevailing context, and rostral regions form more abstract rules to enable long-term control of behavior (Abdallah et al., 2022; Fuster, 2015). Rostral regions achieve maturation later than caudal ones (Dumontheil et al., 2008; Tau and Peterson, 2010; Burgess and Wu, 2013) and this likely contributes to long-lasting and complex goal-directed processes in adulthood. This evidence can be used to design an AGI agent. A possible design for an AGI agent is presented in Figure 1.

The agent can be described as a multi-component system. Input sensorial information is processed by the special sensorial component and influences on external objects are provided by the motor component. These components are multi-layer systems with unchangeable structures, but its detailed descriptions are beyond the scope of the article. It is important to notice that the division of the world into separate objects is not the function of the sensorial component only. The agent also includes the reward/punishment component that provides innate influences on the behavior of the agent. These influences are similar to pain or pleasure being felt by infants. However, the activity of infants cannot be reduced only to the maximization or minimization of such feelings. Similarly, the innate reward/punishment component is involved in the construction of goal-directed processes and provides a particular sort of feedback from the situation; however, the behavior of the agent, in general, is not determined by the criteria being embedded in this component.

The main part of the agent’s design is the goal component, which is multilevel and hierarchical. Each level is a multi-layer neural network with an alterable structure. Levels are distinguished by their threshold of coherence and all layers relating to the given level have the same threshold of coherence. The detailed description of these networks is beyond the scope of the paper, yet it is suggested that owing to a more complex design the threshold of coherence for upper levels is higher than for lower ones. There are feed forward and feedback connections between adjacent levels. The lowermost level is connected with the input and output components of the agent. Due to the different design of the levels, the formation of stable goal-directed processes at level j is more complex and slower than at level j−1. Since the changes in the units associated with ongoing goal-directed processes may be local, information on many processes can be stored and reactivated at each level of the goal component.

Obviously, the development of an AGI agent is distinctive from learning in conventional AI systems. In such systems the researcher is familiar with the design of the intelligent agent, that is, the criteria that are applied to learn the agent in neural networks or the goals that the agent should be capable of achieving in symbolic processing. The researcher is able directly to alter the design if the performance of the agent is inappropriate. However, the structure of an AGI agent, in general, is not transparent to the researcher; therefore, the development of the agent would be similar to nurturing humans. The tutor immerses the agent in various situations and on the basis of the agent’s responses, the tutor changes the agent’s actions.

The development of an AGI agent can be described as follows. Initially the units in the goal component have a low coherence and there are no goal-directed processes. The tutor creates simple situations and goal-directed processes are performed at level 1. After performing various processes at level 1, the tutor concludes that the agent is capable of functioning under such simple conditions efficiently. Then the tutor immerses the agent in novel environments that are a little more complex. This limited complexity of new environments and accumulated skills may lead to goal-directed processes at level 2. Such processes are more long-term and persistent than processes at level 1 because they have a higher threshold of coherence. The goal component is a hierarchical system; therefore, processes at level 2 may activate and inhibit processes at level 1. In a similar vein, level 3 can be achieved, etc. Ultimately, the agent becomes capable of achieving its goals in arbitrary real situations.

This design for an AGI agent can obviously be considered a backbone. Additional components may be necessary. For example, if an AGI agent should function at the human level then the agent should be able to acquire a human-like language and construct symbolic models of the situation. The human brain includes areas that are responsible for acquiring a language and processing verbal information (Luria, 2012). It is reasonable to assume that an AGI agent also should include a language component that has a changeable structure with special layers and separate connections with the motor component.

It is of interest to note that JCA constrains the capabilities of all AGI agents. Indeed, regardless of the computer power of an AGI agent, ineffective actions are possible if the cost of its construction is minimal.

## 4 A simple agent that exhibits goal-directed behavior

**Figure 2:**
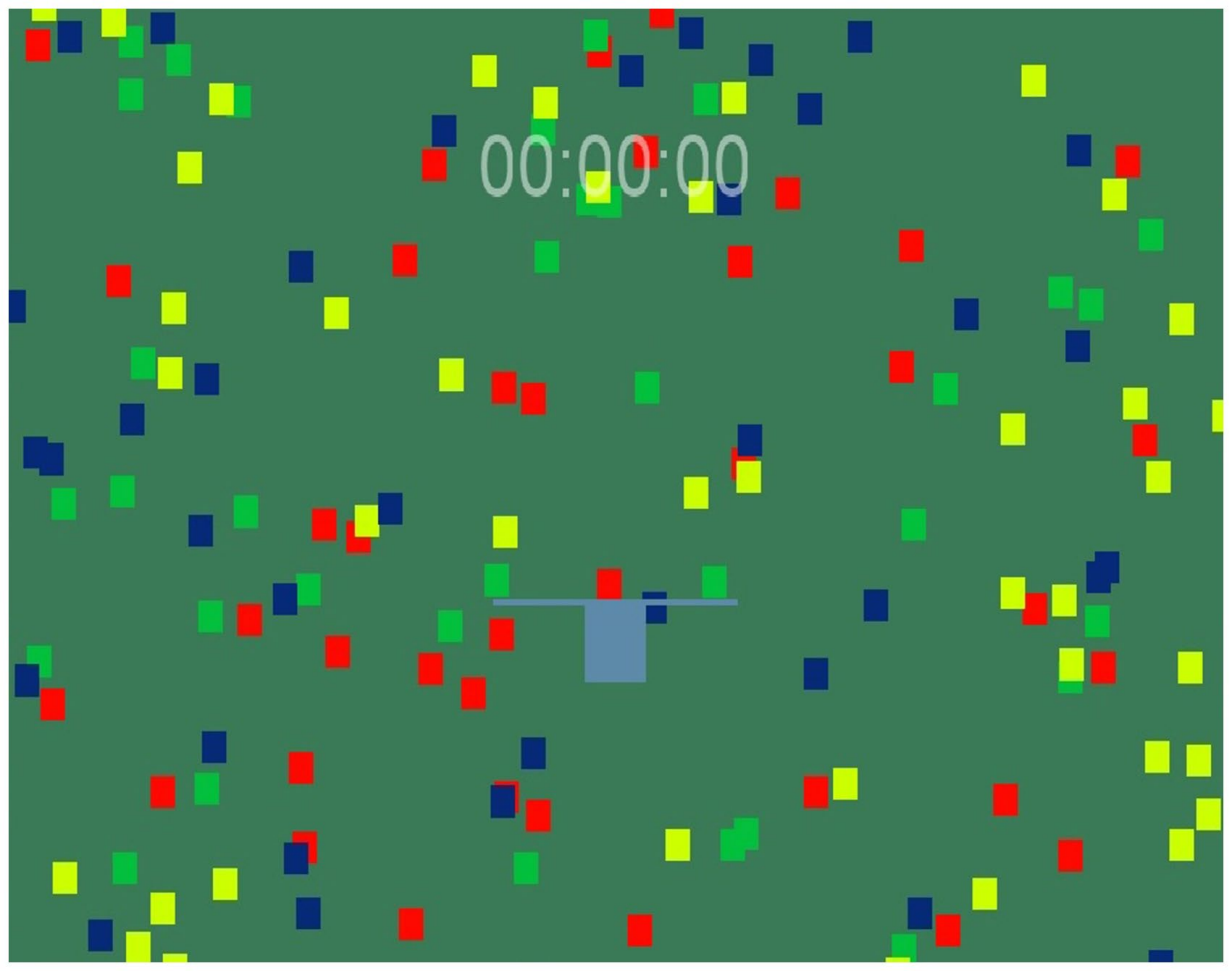
The organism and objects.

In this chapter a model that demonstrates how the joint construction approach can be implemented is presented. The model consists of a quadrangular field filled with different objects and an organism that is capable of moving in discrete time (Figure 2).

There are four sorts of objects being distinguished by their colors. The organism “perceives” several objects simultaneously and then moves to or from these objects depending on their colors. If the organism touches an object, the object disappears and some changes in the organism occur. These changes force the organism to approach some objects and avoid other ones. If the center of the organism reaches the border of the field, the organism leaps to a random position that is on a straight line connecting the point where the center reaches the border and the starting point.

It is obvious that the organism exists in a complex environment when it can be concurrently stimulated to move in several directions and, as a result, it cannot move at all. To some extent, the organism is similar to Buridan’s Ass. The objective of the model was to study how JCA can influence the behavior of the organism under such conditions.

### 4.1 Description of the organism

**Figure 3:**
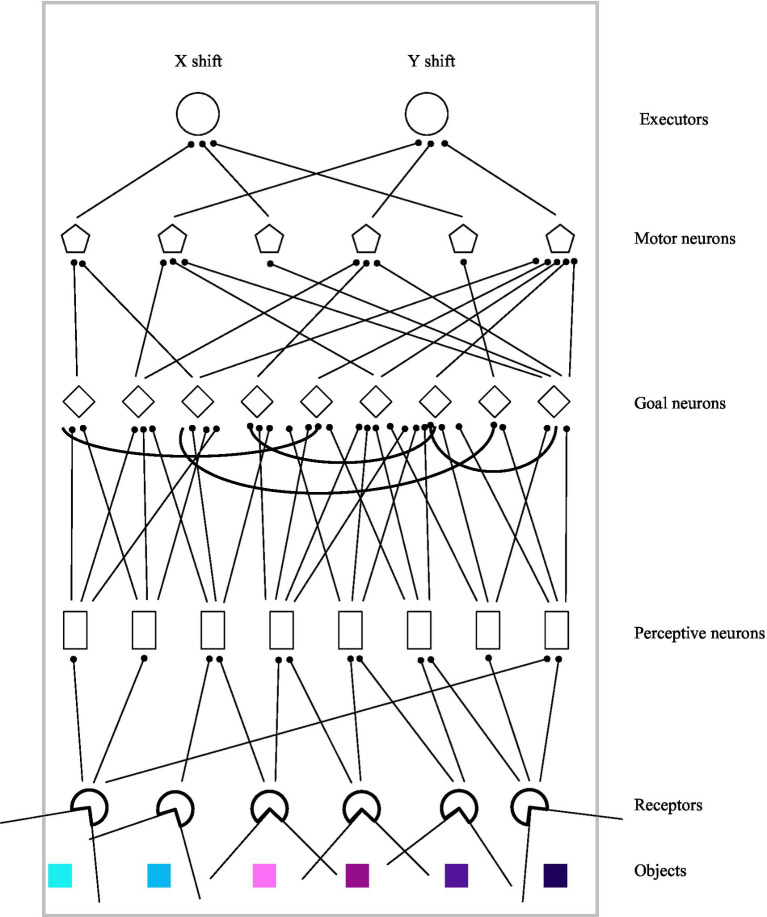
Design of the organism.

The organism is a neural network and in terms of the previous chapter, its goal component includes only one level with one layer. The organism can function as a system having an unchangeable or changeable structure depending on some parameters. It is suggested that if the structure is changeable, goal-directed processes underpin the organism’s behavior. The design of the organism is presented in Figure 3.

Figure 2 displays that the organism has two whiskers. The whiskers constrain the organism’s field of vision. The field of vision can be variable but the field of vision equals to π radians was used in the simulations only. The organism perceives objects by means of receptors. The field of the vision of each receptor is the field of the vision of the organism divided by the number of receptors. If there are several objects in the field of the vision of a receptor then the receptor sends information on the nearest object. Each receptor transmits a Euclidean distance between the organism and the object (D_o_), an angle between the organism and the object (A_o_) and the color of the object. The color is a negative or positive number (color-number, hereinafter). The distance and the angle are normalized:


$$D_{o}=1/\left(1+D_{o}\right),A_{o}=1/\left(1+A_{o}\right).$$

Each perceptive neuron is connected with NC receptors in a topographic fashion. Consequently, neighboring receptors project to neighboring perceptive neurons. NC is the number of connections between one unit of the organism and other units. The number of perceptive neurons is greater than the number of receptors. The input of perceptive neuron j at time t is defined as follows:


$$\begin{array}{l} {\mathrm{Input}}_{j}\left(t\right)=\sum^{\mathrm{Nc}}{w_{i,j}}^{\mathrm{col}}*{{\mathrm{rec}}_{i}}^{\mathrm{col}}\left(t\right)+\sum^{\mathrm{Nc}}{w_{i,j}}^{\mathrm{dist}}*{{\mathrm{rec}}_{i}}^{\mathrm{dist}}\left(t\right)+\\ \;\;\;\;\;\;\;\;\;\;\;\;\;\;\;\;\;\;\;\;\;\;\;\;\;\;\;\;\sum^{\mathrm{Nc}}{w_{i,j}}^{\mathrm{ang}}*{{\mathrm{reec}}_{i}}^{\mathrm{ang}}\left(t\right), \end{array}$$

where col, dist, ang are the color-number, distance and angle of the object perceived by receptor i, w_i,j_ is the weight of the connection between receptor i and perceptive neuron j. All such weights are random numbers in a range from 0 to 0.5. The output of perceptive neuron j is as follows:


$$\mathrm{Output}{\left(t\right)}_{j}=2/\left(1+{e^{-\mathrm{Input}\left(t\right)}}_{j}\right)-1$$

Initially, each goal neuron is connected with NC perceptive neurons in a topographic fashion. The number of goal neurons is greater than the number of perceptive neurons. Moreover, each goal neuron initially is connected with other NC goal neurons that are selected randomly. The input of goal neuron m at time t is as follows:


$${\mathrm{Input}}_{m}\left(t\right)=\sum w_{j,m}*{\mathrm{Output}}_{j}\;\left(t\right)+\sum w_{!m,m}*{\mathrm{Output}}_{!m}\;\left(t-1\right),$$

where w_j,m_ is the weight of the connection between perceptive neuron j and goal neuron m, w_!m,m_ is the weight of the connection between goal neuron m and another goal neuron!m. Thus, the layer of goal neurons is a recurrent neural net. Initially, all weights between perceptive and goal neurons are random numbers in a range from 0 to 0.5 and all weights between goal neurons are random numbers in a range from −0.5 to 0.5. The output of goal neuron m is as follows:


$$\mathrm{Output}{\left(t\right)}_{m}=2/\left(1+{e^{-\mathrm{Input}\left(t\right)}}_{m}\right)-1$$

Initially, each motor neuron is connected with NC goal neurons that are selected randomly. The number of motor neurons is fewer than the number of goal neurons and even. The input of motor neuron n at time t is as follows:


$${\mathrm{Input}}_{n}\;\left(t\right)=\sum w_{m,n}*{\mathrm{Output}}_{m}\;\left(t\right),$$

where w_m,n_ is the weight of the connection between goal neuron m and motor neuron n. Initially, all weights between goal and motor neurons are random numbers in a range from 0 to 0.5. The output of motor neuron n is as follows:


$$\mathrm{Output}{\left(t\right)}_{n}=2/\left(1+{e^{-\mathrm{Input}\left(t\right)}}_{n}\right)-1$$

All motor neurons with odd numbers (2n*−*1) are connected with ExecutorX which moves the organism along X-axis at a distance of DX and all motor neurons with even numbers (2n) are connected with ExecutorY which moves the organism along Y-axis at a distance of DY:


$$\begin{array}{l} \mathrm{DX}=2*k_{\mathrm{dx}}*\left(\begin{array}{l} \sum ™\mathrm{Output}\\ {\left(t\right)}_{\mathrm{2n}-1} \end{array}\right)/\mathrm{TM}\;\\ \mathrm{DY}=2*k_{\mathrm{dy}}*\left(\begin{array}{l} \sum ™\mathrm{Output}\\ {\left(t\right)}_{\mathrm{2n}} \end{array}\right)/\mathrm{TM}, \end{array}$$

where k_dx_ and k_dy_ are coefficients and TM is the number of motor neurons.

All weights between the layers of the organism are positive, initially; therefore if the color-number of an object is positive then the organism initially approaches the object: otherwise, it moves away from the object. The velocity of movement is proportional to the modulus of the color-number.

If the organism perceives no objects, it starts rotating until an object is perceived. As is pointed out above, there are four sorts of objects. Red, green and yellow objects have positive and different color-numbers and blue objects have a negative color-number. If the distance between the center of the organism and an object is less than the size of the organism, this indicates the organism touches the object and the object vanishes. The organism touches objects only if these objects are within its field of vision. Touching blue and yellow objects does not influence the state of the organism. If the organism touches a green object, then for goal neuron m:


$$\begin{array}{l} \mathrm{if}|\mathrm{Output}{\left(t\right)}_{m}|>\mathrm{Tact then}\;w_{j,m}=w_{j,m}*\left(1+k1*{\mathrm{colr}}_{m}\right);\\ w_{m,n}=w_{m,n}*\left(1+k2*{\mathrm{colr}}_{m}\right). \end{array}$$

For a red object the formulas are as follows:


$$\begin{array}{l} \mathrm{if}|\mathrm{Output}{\left(t\right)}_{m}|>\mathrm{Tact then}\;w_{j,m}=w_{j,m}*\left(1+k1*{\mathrm{colr}}_{m}\right);\\ w_{m,n}=w_{m,n}*\left(-1-k2*{\mathrm{colr}}_{m}\right), \end{array}$$

where w_j,m_ and w_m,n_ are the weights of the connections between goal neuron m and perceptive neuron j and motor neuron n, accordingly. Tact, k1, k2, colr_m_ are parameters. Colr_m_ is used to prevent the overflow of weights; therefore, after each change of weights colr_m_ is decreased by multiplication by a number that is less than 1.

It can be concluded from these formulas that if the organism touches a green object this stimulates the organism to approach green objects. The touch of a red object stimulates the avoidance of red objects.

In the model *λ* (t) is formulated as follows:


$$\lambda \;\left(t\right)=\left(\sum^{\mathrm{TGN}}\;{\left({\mathrm{Output}}_{m}\left(t\right)-{\mathrm{Output}}_{m}\left(t-1\right)\right)}^{2}\right)/\mathrm{TGN},$$

where TGN is the total number of goal neurons. Two parameters, designated as T1 and T2 (T1 < T2) determine changes in the goal layer.

If λ(t) < T1 this indicates the situation is unaltered. In this case, there are no changes in the goal layer. If T1 < λ (t) < T2 this indicates that the situation is changed to some extent. The change in the situation is useful for leaving possible traps and therefore such a state of the organism is reinforced. A simple gradient descent algorithm is used for this as follows:


$$\begin{array}{l} w_{j,m}=w_{j,m}+\mathrm{KB}*{\mathrm{Output}}_{j}*0.5*\;\left({\mathrm{Output}}_{m}\;\left(t\right)-{\mathrm{Output}}_{m}\left(t-1\right)\right)*\\ \;\;\;\;\;\;\;\;\;\;\;\;\;\;\;\;\;\;(1+{\mathrm{Output}}_{m}\left(t\right)*\left(1-{\mathrm{Output}}_{m}\left(t\right)\right). \end{array}$$

$$\begin{array}{l} w_{!m,m}=w_{m!,m}+\mathrm{KB}*{\mathrm{Output}}_{!m}*0.5*\;\left(\begin{array}{l} {\mathrm{Output}}_{m}\;\left(t\right)-\\ {\mathrm{Output}}_{m}\left(t-1\right) \end{array}\right)*\\ \;\;\;\;\;\;\;\;\;\;\;\;\;\;\;\;\;\;\;\;\;\;(1+{\mathrm{Output}}_{m}\left(t\right)*\left(1-{\mathrm{Output}}_{m}\left(t\right)\right), \end{array}$$

where w_j,m_ is the weight of the connection between perceptive neuron j and goal neuron m, w_!m,m_ is the weight of the connection between goal neuron m and another goal neuron!m, and KB is a coefficient.

If λ(t) > T2 this indicates that the situation is altered considerably and a new goal-directed process is necessary to respond to such changes. The construction of a novel process corresponds to Equation (1) and includes the generation of novel goal neurons.

Let us introduce the following designations:

ρ(gn_i_) = (Output_i_(t)-Output_i_(t-1))^2^ is a metric of goal neuron i in the space of goal neurons.

gn(i).conI_m_ is input connection m of goal neuron i; gn(i).conIW_m_ is the weight of this connection; len(gn(i).conI) is the number of input connections.

gn(i).conO_m_ is output connection m of goal neuron i; gn(i).conOW_m_ is the weight of this connection; len(gn(i).conO) is the number of output connections. gn.t is a parameter; df is a constant; rand(0,b) is a random number in a range from 0 to b.

The pseudo-code for Equation (1) can be described as follows:

  ζ =0;

  While ζ < Τ do

  Find  goal neuron 1 and  goal neuron 2 for which ρ(gn) are minimal and ρ(1)< ρ(2);

  Create a novel goal neuron, neuron + ;

  for a=1 to len(1.conI) do

   if rand(0, 1)<p (p>0.5)

    gn(+).conI_a_= gn(1).conI_a_ ; gn(+).conIW_a_= gn(1).conIW_a_; gn(1).t=gn(1).t+ df ;ζ =ζ+gn(1).t;

   else

    gn(+).conI_a_= gn(2).conI_a_ ; gn(+).conIW_a_= gn(2).conIW_a_; gn(2).t= gn(2).t+df ; ζ =ζ+gn(2).t;

  for a=1 to len(1.conO) do

   if rand(0, 1)<p

      gn(+).conO_a_= gn(1).conO_a_ ;  gn(+).conOW_a_= gn(1).conOW_a_;

       else

     gn(+).ConO_a_=random motor neuron; gn(+).conOW_a_=rand(0,0.5); Add neuron+ to the goal neuron layer; ρ(+)=(Output_+_(t)-Output_1_(t-1))**2

  end

The minimization of *Λ*() follows from the fact that only two neurons c minimal ρ() interact and participate in the creation of a novel neuron. The minimization of *Ξ*() is associated with gn().t. This parameter reflects the “memory” of a goal neuron regarding its participation in the creation of novel neurons. The higher this parameter in the neurons that are involved in interactions, the fewer novel neurons are created. Since each novel goal neuron gets its components from other goal neurons, one may say that each novel neuron increases the coherence in the layer of goal neurons.

After all possible changes, the goal layer determines the movement of the organism again. Since the organism has only one goal level, the criterion for the achievement of a goal is not formulated in the model.

### 4.2 Simulations and discussion

It is easy to notice that the organism is able to function in four modes of learning depending on the values of three parameters. The term “learning” stands for any changes in the state or the structure of the organism. If Tact, T1, and T2 are very large then the organism is not capable of learning at all. This is Mode 1. In Mode 2, when Tact is less than an empirical threshold and T1 and T2 are very large, the weights between neurons are changed only if the organism touches a red or green object. In Mode 3, when Tact, T1 are less than empirical thresholds and T2 is very large, the weights can additionally be changed by the gradient descent algorithm. Obviously, Mode 2 and Mode 3 can be considered the versions of conventional learning with reinforcement when the structure of the organism is unchangeable. In Mode 4 Tact, T1,T2 are less than empirical thresholds; therefore, the structure of the organism is changeable in this mode.

A typical behavior of the organism in Modes 1, 2, and 4 is presented in Supplementary Videos 1–3. The behavior in Mode 3 is similar to that in Mode 2.

There usually are many objects around the organism and due to the organism’s design and the initial randomness of connections between neurons, the organism may easily be trapped. When the organism is trapped, owing to negative weights between goal neurons the organism usually repeats movements within a limited area. Such a behavior is presented in Supplementary Video 1. Learning can be considered a way of decreasing the randomness in the state of the organism owing to interactions with the environment and therefore learning may allow the organism to leave possible traps. There are three modes in which the state of the organism can be altered and it can be suggested that each of these modes influences how the organism leaves traps. These ideas can be used as a prerequisite for three hypotheses. First, it is necessary to examine whether the organism may be trapped. The second hypothesis suggests the organism is capable of learning in three modes. The third hypothesis suggests Mode 4 is most suitable for leaving possible traps because in this mode there may be the coherence in the functioning of goal neurons. This may benefit the organism to select a direction of movement.

The number of disappeared objects can be used as an estimate of the organism’s activity. In this case, the first hypothesis is that the total number of disappeared objects is considerably fewer than the total number of objects in Mode 1. The second hypothesis expects the number of red disappeared objects is fewer than the number of the disappeared objects of the other colors in three modes because the organism can be learned to avoid red objects. Moreover, the number of green disappeared objects may be greater than the number of blue and yellow disappeared objects in three modes. The third hypothesis suggests that the total number of disappeared objects in Mode 4 is greater than that in the other modes.

The model was coded in Python and run on a standard PC. There were 50 simulations for each mode. In these simulations there were 50 objects of each color that were located randomly. The color-numbers for red, green, yellow, and blue objects were 8, 2, 1, and −1, respectively. In each simulation the organism performed for 15 min. In preliminary probes it was found that this interval of time was sufficient to reveal all possible effects associated with the modes and the colors definitely. In Mode 4 the initial number of goal neurons was 28 but the number of goal neurons tended to increase over time and sometimes this number was about 2000. In preliminary tests it was found the number of goal neurons influenced the behavior of the organism weakly, if this amount is constant. Nevertheless, to minimize the possible effect of the number of goal neurons, this parameter was 1,000 in Modes 1–3. The values of the other parameters were identical for all modes (the list of parameters is in Appendix). Of course, the values for Tact, T1, T2 were used in Mode 4 only. In other modes the values, that were necessary for the given mode were applied. The results of simulations are presented in Table 2.

**Table 2:** An average number of disappeared objects for four modes and four colors.

	Color				
Mode	Red	Green	Blue	Yellow	Total
Mode 1	4.65	4.53	4.05	5.02	18.24
Mode 2	1.98	2.5	2.74	2.8	10.02
Mode 3	1.94	2.9	3.22	2.38	10.44
Mode 4	13.74	15.72	16.12	15.78	61.36

There were eight receptors and the organism usually perceived 5–6 objects simultaneously. Table 2 shows that the organism was really similar to Buridan’s Ass because in Mode 1 the organism touched less than 10 percent of possible objects. The Friedman ANOVA analysis demonstrates that there was not a significant distinction between the number of the disappeared objects of different colors in Mode 1 (*p* = 0.32). Table 2 also shows that in three modes the organism was capable of learning. Indeed, in these modes red objects disappeared significantly less than other objects when compared on the Wilcoxon matched pairs test (all *p*s < 0.05), although the color-number of red objects was positive. There were no significant distinctions between the pairs of other colors in these modes. This indicates that the organism was not learned to approach green objects, probably owing to a minor difference between the color-number of green objects and the color-numbers of blue and yellow ones. Table 2 shows that in Mode 4 the number of vanished objects was several times greater than in the other modes. This confirms the third hypothesis.

The results of the simulations show the organism is capable of learning in Mode 2 and Mode 3, however, those conventional methods for the establishment of the goals-means correspondence were not efficient for leaving traps. On the contrary, since in Mode 1 the total number of disappeared objects was greater than that in Mode 2 and Mode 3, the methods make leaving traps less probable. It is reasonable to suggest that there may be a sort of reinforcement that may be appropriate for the withdrawal from a trap. For example, the organism can be rewarded if it left the trap. However, the definition of a trap is necessary in this case; therefore, such a sort of reinforcement is of limited use. In Mode 4 the organism was capable of leaving traps efficiently despite the organism’s initial random structure. It is important to note that although the innate characteristics associated with the colors of objects influenced the behavior of the organism in Mode 4, these characteristics were not the main determinants of the organism’s actions.

Оne may say that 50 simulations are insufficient to reveal real differences between modes. Such a proposal does not seem correct. There is a huge difference between the average number of disappeared objects in Mode 3 and Mode 4 (61.36 versus 10.44). This difference is highly significant, according to the T-criterion: t(98) = 15.41, *p* = 0.0, and Cohen’s d = 3.08. Since pseudo-random numbers are the only source of variability in the model, it is unclear why the addition of new simulations can alter the difference. The increase in the number of simulations simply makes the difference between the performance of the organism in Mode 3 and Mode 4 even more discernible. Probably the change in the number of simulations can affect other differences, for example, the difference between the performance in Mode 2 and Mode 3; however, such differences are not relevant for the investigation of the role of joint construction.

Another important problem is the sensitivity of the model to changes in its parameters, especially such as Tact, T1 and T2. Preliminary trials demonstrated changes in Tact and T1 affect the model weakly. If Tact is very small, the organism cannot learn to avoid red objects, but an increase in Tact causes the organism to evade red objects. Changes in T1 practically do not influence performance. The considerable increase in T2, of course, makes the behavior of the organism in Mode 4 similar to that in other modes; however, relatively small changes in T2 do not affect the behavior in Mode 4 because the generation of new goal neurons stays possible.

Since the goals that the organism may achieve are not described explicitly and the state of the organism when a goal is achieved is not formulated, one may claim that the behavior of the organism is not goal-directed in Mode 4. Simply, the organism acquires the ability to walk through potential traps. Indeed, the behavior of an agent can be characterized as goal-directed if it is clear that the agent repeatedly attempts to achieve some state despite changes in the situation. It does not appear that the behavior of the organism, when it wanders through the field, corresponds to such a criterion.

However, the organism is able to exhibit another sort of behavior. If the organism, when it is trapped in an area for a long time, is manually moved to another position, then in Modes 1–3 the organism starts moving chaotically until it is trapped in a new area. Such behavior is presented in Supplementary Videos 4, 5. The organism becomes red while it is being forced to move.

In Mode 4 the organism sometimes comes to its trap back and does it fast. Such behavior is presented in Supplementary Videos 6, 7.

The organism exhibits such behaviors in about 10 percent of simulations, given that the duration of a simulation is longer than 10 min and the number of goal neurons exceeds 1,500. If the organism is forcefully moved from the area at early moments, then the organism does not come back and simply wanders through new traps. It is not clear how the organism finds its way back. Since the organism is capable of coming back after long roaming, it can be hypothesized that the organism has a map of the field and somehow uses it. However, if the organism that is capable of coming back in Mode 4 is converted in another mode while keeping its other characteristics invariable, it loses this capability. Thus, the generation of novel goal neurons rather than the map of the field is important for the capability of coming back.

Obviously, coming back is a goal-directed behavior. One can say that if the organism does not leave an area for a long time, then the organism sometimes “decides” that its aim is to stay in this area. It can be hypothesized that a coherent structure emerging in such cases is distributed over the goal layer very widely. As a result, this structure determines the construction of the novel goal-directed processes that arise after moving the organism to another area manually, and such processes become the means that brings the organism back. Since leaving traps is based on the same mechanism as coming back, leaving traps in Mode 4 is a goal-directed behavior.

There may be two sorts of goal-directed behaviors. One of these sorts is a behavior being aimed at the achievement of some changes in the agent and/or the environment. The other sort is a behavior aimed at the maintenance of the current state in the agent and/or the environment. The simulations display that an agent with one goal level performs both sorts of goal-directed behaviors. It is reasonable to assume that an agent with multiple goal levels may be capable of combining both sorts to achieve multi-stage goals in more complex environments.

The model demonstrates that the joint construction approach allows constructing goal-directed processes with arbitrary goals and means. Of course, other methods for the construction of goal-directed processes are possible and may be efficient. For example, in the model, ρ() is a metric in an absolutely abstract space and therefore only two neurons interact at any moment. However, it is possible that ρ() should take the topology of the network into account. In this case, many local minima of ρ() are possible and several neurons may interact with each other in each minimum concurrently. In the model, a novel neuron arises from the interaction. Although new neurons arise in the adult brain (Denoth-Lippuner and Jessberger, 2021), this is a rare event. The emergence of new connections seems more physiologically plausible, especially if several neurons interact jointly. It is possible that the number of neurons involved in the interaction may determine what components of the network (neurons or connections) should be altered. These ideas can also be applicable for multilevel agents.

It is critical to note the given model is absolutely illustrative; the objective of its creation was simply to show that the implementation of the joint construction approach is possible and feasible. The results of the simulation demonstrate the organism exhibits various sorts of goal-directed behavior. This is a necessary prerequisite for more realistic models and, finally, for the creation of agents that can function in real environments. Of course, the advance from a simple model to real AGI agents should be a very complex process, including many trials and errors. The conventional approaches have been developed for decades but agents and systems based on these approaches mostly function in artificial environments. It is reasonable to assume that the development of agents based on JCA can be more successful owing to its adequate theoretical basis.

## 5 Conclusion

Humans are goal-directed agents; therefore; AGI can be constructed following the fundamental principle of the goals-means correspondence. There may be different architectures regarding how the goals-means correspondence can be implemented in the architecture of goal-directed agents. A conventional view that is based on the observations of animals, humans and self-reports suggests there are two goal-directed architectures. However, the conventional architectures cannot explain the purposefulness and flexibility of actions and some characteristics of thinking. The formal analysis of possible architectures displays that there may be another architecture in that arbitrary goals and means are constructed jointly. I posit the joint construction of a goal and a means underlies human goal-directed processes. The idea of the joint construction of goals and means explains the flexibility of human actions and the characteristics of thinking.

The view on humans as intelligent agents that are based on the joint construction of goals and means allows achieving artificial general intelligence. An AGI agent has no basic goals and means; therefore, an AGI agent constructs goal-directed processes by making its structure more coherent. The development of an AGI agent should be gradual, from short and simple goal-directed processes to long and complex ones; hence an AGI agent may be a multilevel system with different thresholds of coherence.

The joint construction approach is used in a model including a simple agent based on a neural network. The model demonstrates that JCA allows the agent to adapt to the complex environment more efficiently than alternative approaches because when JCA is enabled, the behavior of the agent can be characterized as goal-directed.

## Data Availability

The original contributions presented in the study are included in the article/Supplementary material, further inquiries can be directed to the corresponding author.

## Supplementary material

SUPPLEMENTARY VIDEO 1 Typical behavior in Mode 1.

SUPPLEMENTARY VIDEO 2 Typical behavior in Mode 2.

SUPPLEMENTARY VIDEO 3 Typical behavior in Mode 4.

SUPPLEMENTARY VIDEO 4 Typical forced behavior in Mode 2.

SUPPLEMENTARY VIDEO 5 Typical forced behavior in Mode 3.

SUPPLEMENTARY VIDEO 6 Example of forced behavior in Mode 4.

SUPPLEMENTARY VIDEO 7 Another example of forced behavior in Mode 4.

## Appendix

The number of receptors is 8. The number of perceptive neurons is 16. The number of motor neurons is 20. NC = 5, k_dx_ = k_dy_ = 20, Tact = 0.1, k1 = k2 = 0.3. KB = 100, T1 = 0.01, T2 = 0.05, *p* = 0.7, Τ = 1, df = 0.01.

## References

[ref1] Abdallah M. Zanitti G. E. Iovene V. Wassermann D. (2022). Functional gradients in the human lateral prefrontal cortex revealed by a comprehensive coordinate-based meta-analysis. eLife 11:e76926. doi: 10.7554/eLife.76926, PMID: 36169404 PMC9578708

[ref2] Bago B. De Neys W. (2017). Fast logic?: examining the time course assumption of dual process theory. Cognition 158, 90–109. doi: 10.1016/j.cognition.2016.10.014, PMID: 27816844

[ref3] Bang Y. Cahyawijaya S. Lee N. Dai W. Su D. Wilie B.. (2023). A multitask, multilingual, multimodal evaluation of chatgpt on reasoning, hallucination, and interactivity. *arXiv* [Preprint]. *arXiv:2302.04023*. doi: 10.48550/arXiv.2302.04023

[ref4] Bertino E. Piero G. Zarria B. C. (2001). Intelligent database systems. Reading, MA: Addison-Wesley Professional.

[ref5] Bruckmaier G. Krauss S. Binder K. Hilbert S. Brunner M. (2021). Tversky and Kahneman’s cognitive illusions: who can solve them, and why? Front. Psychol. 12:584689. doi: 10.3389/fpsyg.2021.584689, PMID: 33912097 PMC8075297

[ref6] Buller D. J. (1999). “DeFreuding evolutionary psychology: Adaptation and human motivation” in Where biology meets psychology: Philosophical essays. ed. Hardcastle V. G. (Cambridge, MA: MIT Press), 99–114.

[ref7] Burgess P. W. Wu H. (2013). Rostral prefrontal cortex (Brodmann area 10). In Stuss D. T. Knigh R. T.t (eds), Principles of frontal lobe function, 524–544, Oxford, Oxford University Press

[ref8] Crevier D. (1993). AI: The tumultuous search for artificial intelligence. New York, NY: BasicBooks.

[ref9] Deary I. J. Penke L. Johnson W. (2010). The neuroscience of human intelligence differences. Nat. Rev. Neurosci. 11, 201–211. doi: 10.1038/nrn2793, PMID: 20145623

[ref10] Denoth-Lippuner A. Jessberger S. (2021). Formation and integration of new neurons in the adult hippocampus. Nat. Rev. Neurosci. 22, 223–236. doi: 10.1038/s41583-021-00433-z, PMID: 33633402

[ref11] Devlin J. Chang M. W. Lee K. Toutanova K. (2018). Bert: Pre-training of deep bidirectional transformers for language understanding. *arXiv* [Preprint]. *arXiv:1810.04805*. doi: 10.48550/arXiv.1810.04805

[ref12] Dijksterhuis A. Nordgren L. F. (2006). A theory of unconscious thought. Perspect. Psychol. Sci. 1, 95–109. doi: 10.1111/j.1745-6916.2006.00007.x, PMID: 26151465

[ref13] Dumontheil I. Burgess P. W. Blakemore S. J. (2008). Development of rostral prefrontal cortex and cognitive and behavioural disorders. Dev. Med. Child Neurol. 50, 168–181. doi: 10.1111/j.1469-8749.2008.02026.x, PMID: 18190537 PMC2488407

[ref14] Evans J. S. B. T. (2003). In two minds: dual-process accounts of reasoning. Trends Cogn. Sci. 7, 454–459. doi: 10.1016/j.tics.2003.08.012 14550493

[ref15] Evans J. S. B. T. (2008). Dual-processes accounts of reasoning. Annu. Rev. Psychol. 59, 255–278. doi: 10.1146/annurev.psych.59.103006.093629, PMID: 18154502

[ref16] Evans J. S. B. Stanovich K. E. (2013). Dual-process theories of higher cognition: advancing the debate. Perspect. Psychol. Sci. 8, 223–241. doi: 10.1177/1745691612460685, PMID: 26172965

[ref17] Fjelland R. (2020). Why general artificial intelligence will not be realized. Humanit. Soc. Sci. Commun. 7, 1–9. doi: 10.1057/s41599-020-0494-4

[ref18] Fuster J. (2015). The prefrontal cortex. Cambridge, MA: Academic Press.

[ref19] Jackson P. (1998). Introduction to expert systems. Addison-Wesley Longman Publishing Co.

[ref20] Kahneman D. (2011). Thinking, fast and slow. New York, NY: Farrar, Straus and Giroux.

[ref21] Krizhevsky A. Sutskever I. Hinton G. E. (2017). Imagenet classification with deep convolutional neural networks. Commun. ACM 60, 84–90. doi: 10.1145/3065386

[ref22] Landau L. D. Lifshitz E. M. (1976). Mechanics, course of theoretical physics. Oxford: Butterworth-Heinenann.

[ref23] Lee M. (2023). A mathematical interpretation of autoregressive generative pre-trained transformer and self-supervised learning. Mathematics 11:2451. doi: 10.3390/math11112451

[ref24] Levy D. (2013). Computer chess compendium. Heidelberg: Springer Science and Business Media.

[ref25] Lewkowycz A. Andreassen A. Dohan D. Dyer E. Michalewski H. Ramasesh V.. (2022). Solving quantitative reasoning problems with language models. *arXiv* [Preprint]. *arXiv:2206.14858.* doi: 10.48550/arXiv.2206.14858

[ref26] Luria A. R. (2012). Higher cortical functions in man. Heidelberg: Springer Science and Business Media.

[ref27] Matsuo Y. LeCun Y. Sahani M. Precup D. Silver D. Sugiyama M.. (2022). Deep learning, reinforcement learning, and world models. Neural Netw. 152, 267–275. doi: 10.1016/j.neunet.2022.03.037, PMID: 35569196

[ref28] Meyer A. Frederick S. (2023). The formation and revision of intuitions. Cognition 240:105380. doi: 10.1016/j.cognition.2023.105380, PMID: 37659355

[ref29] Newell A. Simon H. A. (1972). Human problem solving (vol. 104). Englewood Cliffs, NJ: Prentice-Hall.

[ref30] Parker-Holder J. Rajan R. Song X. Biedenkapp A. Miao Y. Eimer T.. (2022). Automated reinforcement learning (autorl): a survey and open problems. J. Artif. Intell. Res. 74, 517–568. doi: 10.1613/jair.1.13596

[ref31] Pennycook G. Trippas D. Handley S. J. Thompson V. A. (2014). Base rates: both neglected and intuitive. J. Exp. Psychol. Learn. Mem. Cogn. 40, 544–554. doi: 10.1037/a0034887, PMID: 24219086

[ref32] Prudkov P. N. (2010). A view on human goal-directed activity and the construction of artificial intelligence. Mind. Mach. 20, 363–383. doi: 10.1007/s11023-010-9218-7, PMID: 40353265

[ref33] Prudkov P. N. (2021). The joint construction of goals and means as a solution for the problem of variability in behavior. Theory Psychol. 31, 480–484. doi: 10.1177/09593543211014957

[ref34] Prudkov P. Rodina O. (2023). A view on the determenistic explanation of actions based on the joint construction of goals and means. Med. Res. Arch. 11, 1–19. doi: 10.18103/mra.v11i12.4802

[ref35] Ren P. Xiao Y. Chang X. Huang P. Y. Li Z. Chen X.. (2021). A comprehensive survey of neural architecture search: challenges and solutions. ACM Comput. Surv. 54, 1–34. doi: 10.1145/3447582, PMID: 39076787

[ref36] Russell S. J. Norvig P. (2009). Artificial intelligence: A modern approach. Upper Saddle River, NJ: Prentice Hall.

[ref37] Silver D. Hubert T. Schrittwieser J. Antonoglou I. Lai M. Guez A.. (2018). A general reinforcement learning algorithm that masters chess, shogi, and go through self-play. Science 362, 1140–1144. doi: 10.1126/science.aar6404, PMID: 30523106

[ref38] Silver D. Singh S. Precup D. Sutton R. S. (2021). Reward is enough. Artif. Intell. 299:103535. doi: 10.1016/j.artint.2021.103535

[ref39] Stanley K. O. Clune J. Lehman J. Miikkulainen R. (2019). Designing neural networks through neuroevolution. Nat. Mach. Intell. 1, 24–35. doi: 10.1038/s42256-018-0006-z

[ref40] Tau G. Z. Peterson B. S. (2010). Normal development of brain circuits. Neuropsychopharmacology 35, 147–168. doi: 10.1038/npp.2009.115, PMID: 19794405 PMC3055433

[ref41] Thompson V. A. Johnson S. C. (2014). Conflict, metacognition, and analytic thinking. Think. Reason. 20, 215–244. doi: 10.1080/13546783.2013.869763

[ref42] Turing A. (1950). Computing machinery and intelligence. Mind 59, 433–460.

[ref43] Vinyals O. Babuschkin I. Czarnecki W. M. Mathieu M. Dudzik A. Chung J.. (2019). Grandmaster level in StarCraft II using multi-agent reinforcement learning. Nature 575, 350–354. doi: 10.1038/s41586-019-1724-z, PMID: 31666705

